# Clinical impact of lifestyle interventions for the prevention of diabetes: an overview of systematic reviews

**DOI:** 10.1136/bmjopen-2016-013806

**Published:** 2016-12-21

**Authors:** Lara Howells, Besma Musaddaq, Ailsa J McKay, Azeem Majeed

**Affiliations:** 1 Department of Primary Care and Public Health, Imperial College London, London, UK; 2 Royal Free Hospital, London, UK

**Keywords:** Diabetes prevention programme, Diet, Physical activity, Intermediate hyperglycaemia

## Abstract

**Objectives:**

To review the clinical outcomes of combined diet and physical activity interventions for populations at high risk of type 2 diabetes.

**Design:**

Overview of systematic reviews (search dates April–December 2015).

**Setting:**

Any level of care; no geographical restriction.

**Participants:**

Adults at high risk of diabetes (as per measures of glycaemia, risk assessment or presence of risk factors).

**Interventions:**

Combined diet and physical activity interventions including ≥2 interactions with a healthcare professional, and ≥12 months follow-up.

**Outcome measures:**

Primary: glycaemia, diabetes incidence. Secondary: behaviour change, measures of adiposity, vascular disease and mortality.

**Results:**

19 recent reviews were identified for inclusion; 5 with AMSTAR scores <8. Most considered only randomised controlled trials (RCTs), and RCTs were the major data source in the remainder. Five trials were included in most reviews. Almost all analyses reported that interventions were associated with net reductions in diabetes incidence, measures of glycaemia and adiposity, at follow-up durations of up to 23 years (typically <6). Small effect sizes and potentially transient effect were reported in some studies, and some reviewers noted that durability of intervention impact was potentially sensitive to duration of intervention and adherence to behaviour change. Behaviour change, vascular disease and mortality outcome data were infrequently reported, and evidence of the impact of intervention on these outcomes was minimal. Evidence for age effect was mixed, and sex and ethnicity effect were little considered.

**Conclusions:**

Relatively long-duration lifestyle interventions can limit or delay progression to diabetes under trial conditions. However, outcomes from more time-limited interventions, and those applied in routine clinical settings, appear more variable, in keeping with the findings of recent pragmatic trials. There is little evidence of intervention impact on vascular outcomes or mortality end points in any context. ‘Real-world’ implementation of lifestyle interventions for diabetes prevention may be expected to lead to modest outcomes.

**Strengths and limitations of this study**

- Our wide, thorough and systematic search identified a large volume of recent work for consideration.
- Our work followed widely accepted methodological standards.
- We did not consider the quality of primary studies in detail but relied on the methodologies of individual systematic reviews.
- We were unable to consider outcomes not addressed in the systematic reviews considered.
- We did not systematically search for recent, relevant primary studies.

## Introduction

Type 2 diabetes mellitus (T2DM) is a chronic disease characterised by insulin resistance and hyperglycaemia, associated with various macrovascular and microvascular complications, reduced quality of life and reduced life expectancy.^1^ The global prevalence of T2DM has risen rapidly over the past three to four decades, driven by similar prevalence trends for overweight/obesity, population ageing and changes to population ethnic composition.^2–4^ In 2014, estimated global diabetes prevalence among adults was 8.5%,^4^ with estimates suggestive that 90% of these cases are T2DM. Global prevalence among adults is predicted to reach 9.9% by 2030,^5^ in line with anticipated upwards trends in risk factors. The costs of diabetes are high. In 2012, 1.5 million deaths were attributed to the disease, and a further 2.2 million to hyperglycaemia.^4^ The lower bound of estimated global expenditure on diabetes for 2014 was 11% of total health expenditure,^6^ and this proportion and absolute spending are anticipated to increase alongside the ongoing upwards trends in disease burden.

From a primary prevention perspective, reducing the burden of disease due to diabetes will require implementation of interventions that will reverse overweight/obesity trends to the extent that this more than offsets the anticipated accumulation of risk predicted by changing population demographics.^7^ In some countries, lifestyle (diet and physical activity) education and supported behaviour change programmes for the population with intermediate hyperglycaemia are currently being considered as part of the solution.^8–10^ Trial data do suggest that such interventions can prevent or delay progression to T2DM.^11–15^ However, there is less evidence for impact on glycaemia outwith formal explanatory trial settings.^16^ There is also currently little evidence that such programmes impact on microvascular and macrovascular outcomes,^15^ ^17^ which is important as complications account for much of the morbidity and mortality associated with diabetes, and more than half of global diabetes expenditure.^18^ There have been many systematic reviews on this topic, and they do not all reach the conclusion that sufficient data are available to recommend the use of diabetes prevention programmes (DPPs) at this time. We have therefore aimed to conduct a systematic overview of these reviews, specifically aiming to address the following questions:

1. Do combined diet and physical activity interventions for those at high risk of diabetes impact on glycaemic control and diabetes incidence (primary outcomes)?
2. Do combined diet and physical activity interventions for those at high risk of diabetes impact on dietary and physical activity behaviours, measures of adiposity, microvascular/macrovascular risk, disease or events, quality-adjusted life years or mortality (secondary outcomes)?
3. Does the effect of combined diet and physical activity interventions on the above outcomes depend on participant age, sex or ethnicity?
4. Does the effect of combined diet and physical activity interventions on the above outcomes depend on the nature of the trial (explanatory vs pragmatic trial)?

## Methods

This overview was conducted according to the relevant aspects of the PRISMA guidance^19^ and the Cochrane Handbook of Systematic Reviews (Chapter 22: Overviews of reviews).^20^ Following scoping searches, a review protocol was developed, describing the search strategy and methods for data collection and analysis.

### Search

The population and intervention elements of the review questions (above) were used to generate search terms (see table 1), in turn used to identify associated subject headings within each database of interest, as relevant. No search terms based on comparators or outcomes were used. Systematic review search filters were chosen based on performance in published analyses (eg, ‘meta-analys:.mp. OR search:.tw. OR review.pt.’ was chosen for use in Embase based on the outcomes reported in ref. 18).^21^ Search strategies were trialled before use, to ensure that relevant articles identified during the scoping search would be returned from each database. Adaptations were made where necessary.

**Table 1:** Search terms

	P	I	C	O	S
Key terms	Intermediate hyperglyc*emia	Lifestyle	No associated terms used in searches		Study design terms drawn from previous studies (see text for details)
Additional terms	Impaired glucose tolerance, glucose tolerance impairment, impaired glucose sensitivity, glucose intolerance, intermediate glyc*emic control, impaired fasting glucose, glucose dysregulation, impaired fasting glyc*emia, pre*diabetes, pre*diabetic, pre*diabetes state, pre*diabetic state, latent diabetes, latent diabetic, borderline diabetes, borderline diabetic, borderline HbA1c, borderline hyperglyc*emia, borderline h*emoglobin A1c, borderline A1c, sub*diabetic hyperglyc*emia, non*diabetic hyperglyc*emia, diabetes prevention	Life*style, non*pharmacological intervention, diet, diet therapy, nutrition, dietetics, dietician, nutritionist, nutrition* counsel*ing, dietary intake, healthy eating, physical activity, exercise, physical conditioning, sport, resistance training, aerobics, work*out, strength training, weight training, prevention, preventive health service, preventative health service, preventive intervention, preventative intervention, prevention programme, prevention programme, risk reduction, harm reduction, behavio*r modification, behavio*r change, behavio*r therapy, diabetes education, health education, health promotion, community*based intervention, community*based programme, community*based programme			

Population and intervention identifiers from research questions (‘key terms’) and database-derived and thesaurus-derived alternatives (‘additional terms’).

*Wildcard character.

The identified search terms were used to search MEDLINE (via PubMed), Embase (via Ovid), Web of Science, The Cochrane Library, Centre for Reviews and Dissemination database, Joanna Briggs Institute database, EPPI-Centre Database of Promoting Health Effectiveness Reviews and CINAHL database between 16 April 2015 and 24 April 2015. The searches were updated between 22 November 2015 and 07 December 2015. An example database search strategy is provided in online supplementary appendix 1. We searched review registries and Open Grey between the same dates. Diabetes Care, Diabetologia and Diabetic Medicine were hand-searched between 17 April 2015 and 27 April 2015, and again on 12 December 2015. The reference lists of included papers were also searched.

10.1136/bmjopen-2016-013806.supp1 supplementary appendices

At this stage, no restrictions were placed on language of publication, publication type or publication status. However, we limited publication dates to post-1990 (no upper limit), in view of the relatively recent interest in lifestyle interventions for diabetes prevention and the need to retain relevance to the current healthcare context.

### Selection

The overview inclusion/exclusion criteria are listed in table 2. We included systematic reviews of structured combined diet and physical activity interventions for adults at high risk of diabetes. High risk of diabetes was defined as impaired glucose tolerance, impaired fasting glucose and/or borderline HbA1c (by any established criteria—subject to variation given the time periods and geographical areas covered by the search), metabolic syndrome, overweight/obesity, presence of multiple cardiovascular risk factors and/or high diabetes-risk or cardiovascular-risk score outcome. The intervention was required to include ≥2 interactions with a healthcare professional. Where additional (ie, non-lifestyle) interventions were investigated, we included the study so long as diet and physical activity interventions were analysed separately. We included studies where interventions with diet and/or physical activity components were analysed together, so long as combined diet and physical activity interventions applied to ≥75% of the number of primary studies included in the analyses of interest, and total n-number relevant to these analyses. Similarly, we required the duration of follow-up to be ≥12 months for ≥75% of the number of studies included in the analyses of interest and total number of participants. Reviews considering single group and comparative studies were included, and we required at least 25 participants per treatment arm of each primary study (or n≥50 for cohort studies)—at follow-up—for ≥75% of reviewed studies. Comparator groups were required to receive no/usual care or a lower intensity lifestyle intervention. Studies were classed as systematic reviews if they met the Centre for Reviews and Dissemination Database of Abstracts of Reviews of Effects criteria (see table 2)^22^ and included a clear statement of the clinical topic, description of evidence retrieval methods and sources, and at least one study that met minimum methodological standards for inclusion—as per additional guidance.^21^ ^23^ Where reviews had been updated, we included the most recent version only. Non-English language studies were excluded during selection, with the number of studies excluded for this reason recorded.

**Table 2:** Inclusion and exclusion criteria

	Inclusion criteria	Exclusion criteria
P	≥18 years High risk of diabetes: impaired glucose tolerance, impaired fasting glucose and/or borderline HbA1c (by any established criteria—subject to variation given the time-periods and geographical areas covered by the search), metabolic syndrome, overweight/obesity, presence of multiple cardiovascular risk factors and/or high diabetes-risk or cardiovascular-risk score outcome n-number for each treatment arm of each primary study ≥25 for ≥75% of included studies	Review limited to study of populations with previous gestational diabetes
I	≥75% of primary studies assess combined diet and physical activity intervention involving ≥2 interactions with a healthcare professional, and ≥75% of total review n-number received such an intervention	Diet or physical activity intervention alone
		No face-to-face or telephone contact with healthcare professional
C	No/usual care or lower intensity intervention (where relevant)	Comparison with pharmacological or surgical intervention only
O	Duration of follow-up ≥12 months for ≥75% of the number of studies and total number of participants	
S	Systematic review as per Centre for Reviews and Dissemination Database of Abstracts of Reviews of Effects criteria,^19^ plus clear statement of the clinical topic, description of evidence retrieval methods and sources, and inclusion of at least one study that met minimum methodological standards for inclusion Reviews considering single group and/or comparative studies included Review published post-1990	Review updated Non-English language review

Two reviewers screened the titles and abstracts of the combined search results. Studies that potentially met the inclusion criteria at this point were subject to full review by two reviewers, with exclusions made according to the criteria above. Discrepancies in study selection were resolved by discussion and consensus decision.

### Data extraction and quality assessment

Extraction of information about review aims, methods and results—including quality of evidence for outcomes presented—was achieved via structured data extraction proforma. The proforma was tested on a subset of papers, with changes made before final use in data collection. Additionally, information about the primary studies included in each review was recorded such that the extent of overlap between the sets of primary studies included in each review could be assessed. The data extraction was carried out by one reviewer and checked by a second reviewer, with discrepancies resolved by discussion and consensus decision. Two reviewers independently assessed the quality of each review using the AMSTAR criteria.^24^ Inconsistencies were resolved by discussion and consensus decision.

### Data synthesis

Narrative synthesis methods were used. The review metadata were first summarised by tabulating the inclusion/exclusion criteria, number of studies and participants involved, outcomes of interest assessed and quality assessment results. A matrix demonstrating the primary studies included in each review—and therefore the number of times each primary study had been included—was also developed.

The characteristics of the participants and interventions considered in each review were then also summarised via tabulation, and tables describing the review findings were produced for each outcome of interest, in turn. The consistency of direction and magnitude of any effect, across the different reviews, was considered for each outcome. Where inconsistencies were identified, the additional study data (ie, review methodology, overall population characteristics, outcome definitions and/or quality of studies) were examined for potential explanatory factors. The outcomes for the prespecified subgroups of interest were assessed in the same way. No exclusions were made on the basis of the study metadata, but these were used to consider the relative weight of each primary study in producing the outcomes reported (ie, the potential impact of inclusion of individual primary studies in multiple reviews), and a sensitivity analysis excluding studies with AMSTAR scores <8 was undertaken for each outcome.

## Results

**Figure 1:**
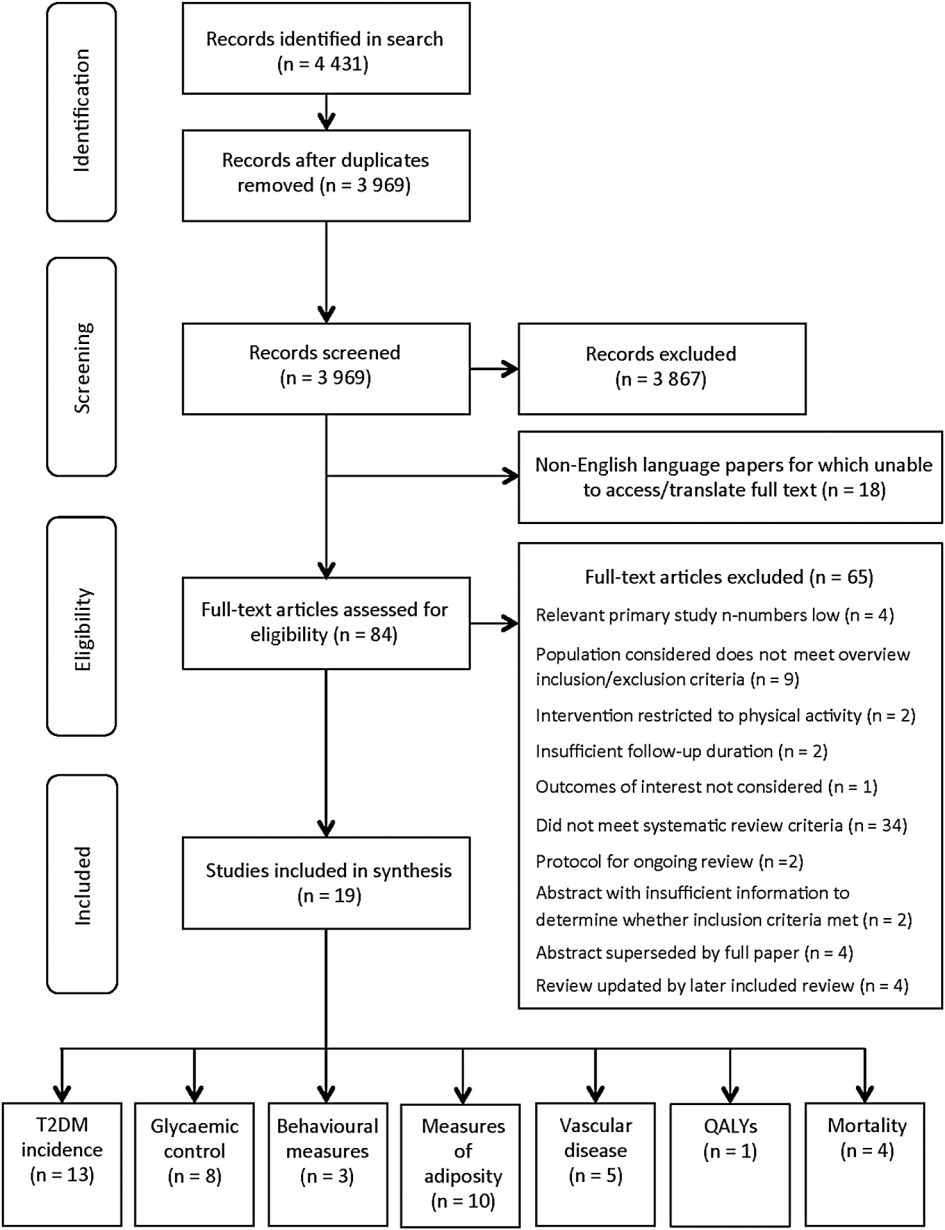
Flow chart demonstrating handling of papers returned by search. Chart adapted from Moher *et al*.^19^ T2DM, type 2 diabetes mellitus; QALYS, quality-adjusted life years.

The search produced 3969 papers for review. Figure 1 displays the handling of the search results. Nineteen studies were selected for inclusion.^1^ ^16^ ^25–41^ A list of the studies excluded at the stage of full-text review is available in online supplementary appendix 2.

### Study metadata

The study metadata are summarised in online supplementary appendix 3. All reviews had been published between 2005 and 2016, and the majority (15/19) post-2010. All except one (Ashra *et al*, 2015)^25^ were peer-reviewed journal articles. There was considerable overlap in primary studies between reviews. In particular, five trials were included in most reviews: the US Diabetes Prevention Programme (DPP), the Finnish Diabetes Prevention Study (DPS), the Indian Diabetes Prevention Programme (IDPP), the Da Qing study and a single-site UK-based trial (Oldroyd *et al*, 2006).^42^ Most reviews (13/19) included only RCTs. RCTs constituted the majority of studies included in all remaining reviews, and in several of these only the RCT data were used in meta-analysis.

Regarding inclusion/exclusion criteria, measures of glycaemia were the most frequently used indicators of high diabetes risk (16/19 reviews). Formal diabetes risk assessment, presence of metabolic syndrome, presence of obesity and/or presence of other T2DM risk factors were also used to indicate high risk (each used in three reviews).

### Quality assessment outcomes

Summary quality assessment results are displayed in online supplementary appendix 3. AMSTAR checklist points were most frequently deducted for failing to state that grey literature had been searched or for omitting to provide a complete list of excluded studies. Five studies had an AMSTAR score of ≤7 and were therefore excluded from sensitivity analyses (see above).^26^ ^30^ ^34^ ^37^ ^40^

### Interventions investigated in reviewed studies

The nature and durations of interventions reviewed varied between studies. Details of the interventions reviewed in each case are summarised in online supplementary appendix 4. Where reported (7/19 reviews), intervention duration ranged from 1 month to 10 years. Programme intensity, extent of group versus individual delivery, intervention settings, methods of delivery and diet and physical activity advice given were variable. Some interventions followed a fixed intensity approach, whereas others used an initial intensive phase, before an intermittent maintenance phase. Most of the physical activity programme components consisted of advice to increase aerobic physical activity, with variable use of structured or unstructured supervised physical activity sessions. Dietary components frequently involved advice on energy restriction, portion control and/or customised dietary counselling.

Where ranges were specified (15/19 reviews), follow-up durations ranged from 4 months to 20 years. In most of these reviews (11/15 cases), the minimum follow-up period was at least 1 year.

### Participant characteristics

Participant characteristics are summarised in online supplementary appendix 4. These relate to all participants in each review, some of which considered pharmacological and/or surgical interventions, as well as lifestyle options. Participant age was reported in all but one review, and variably summarised as a range, range of primary study-level means or overall review mean or median. Ranges extended from 20 to 79 years and mean/medians from 38 to 65 years. Similar summary gender measures were reported (available for 11 reviews), with the proportions of female participants in the reviewed studies (reported in 5 reviews) ranging from 0% to 100%, and overall mean/median per cent female ranging from 34.7% to 66% (reported in five reviews). Summary measures of ethnicity were rarely available, although reviews often reported that samples were of diverse ethnic background. Summary baseline measures of body mass index (BMI) were reported in 10 reviews, with all primary study and review mean values reported >24 kg/m^2^, and the upper limit of primary study means reported being 37.4 kg/m^2^.

### Intervention effects—primary outcomes

#### Diabetes incidence

Incident diabetes was considered in 16 reviews, with reported outcomes drawing on 4–16 primary studies in each case (see table 3). Meta-analyses were run in 12 reviews, and all meta-analysis outcomes were indicative that intervention was associated with lower rates of progression to diabetes, with risk reductions generally in the region of 50–60%. The outcomes of narrative syntheses agreed that intervention was associated with lower progression to diabetes in most studies. These syntheses additionally noted that the effects were more convincing in the larger, more intensive, longer term trials and that intervention effect could be transient.^28^ ^35^ ^40^ One analysis suggested that adherence to lifestyle change might be an important mediator of sustained impact and potentially sensitive to the duration of intervention.^28^ Excluding reviews with AMSTAR scores ≤7 (n=4) had little impact on overall outcomes.

**Table 3:** Incident diabetes and additional glycaemia outcomes

Author, publication date	Number of studies included in syntheses	Outcomes
*Incident diabetes*		
Ashra, 2015	11 studies	Intervention associated with lower rate of progression to diabetes: meta-analysis IRR=0.74 (95% CI 0.58 to 0.93)
*Baker, 2011*	*7 studies*	*Intervention associated with significantly lower incidence of T2DM in all studies reviewed. RR reduction ranged from 29% to 75%.*
Balk, 2015	16 studies	Intervention associated with lower rate of incident diabetes: summary RR=0.59 (95% CI 0.51 to 0.66)
Gillett, 2012	5 systematic reviews; 9 RCTs	Authors observed that lifestyle interventions were associated with lower rates of progression to diabetes in most studies and concluded that some diabetes can be prevented or delayed by lifestyle interventions, with larger, longer term trials (the DPS, DPP and Da Qing study) providing the best evidence. Some evidence that this intervention effect was temporary was also noted, as well as the DPS suggestion that adherence to lifestyle change may be an important mediator of impact on diabetes risk.
Glechner, 2015	5 studies	Meta-analysis outcomes suggest intervention non-significantly associated with lower risk of progression to T2DM at 1 year: RR=0.60 (95% CI 0.35 to 1.05; 4 studies). Evidence of significant intervention effect on progression to diabetes at 3 years: RR=0.63 (0.51 to 0.79; 5 studies).
Hopper, 2011	4 studies	In meta-analysis, intervention associated with lower rate of progression to T2DM: RR=0.52 (95% CI 0.46 to 0.58).
Merlotti, Morabito and Pontiroli, 2014	11 studies	Intervention associated with lower rate of progression to diabetes: meta-analysis OR=0.43 (95% CI 0.35 to 0.52).
Merlotti, Morabito, Ceriani and Pontiroli, 2014	4 studies	Intervention associated with lower rates of progression to diabetes: meta-analysis OR=0.44 (95% CI 0.36 to 0.52).
*Modesti, 2016*	*8 studies*	*Intervention associated with lower rates of progression to diabetes: meta-analysis OR=0.55 (95% CI 0.44 to 0.70).*
Norris, 2005	5 studies	Intervention associated with significantly lower cumulative incidence of diabetes in three of the five trials reviewed (RR reductions=58% (95% CI 48 to 66), 51% and 58%). Trials in which effect observed involved intensive, sustained, multicomponent interventions.
Orozco, 2008	8 studies	Intervention associated with lower rates of progression to diabetes: meta-analysis RR=0.63 (95% CI 0.49 to 0.79). Similar results when largest study (DPP; weight=26%) excluded: RR=0.69 (0.55 to 0.87).
*Santaguida, 2005*	*5 studies*	*Significantly lower rates of progression to diabetes or higher rates of reversion to normal glucose tolerance, observed in intervention vs control arms of 4/5 trials. (NB. Number of studies considering progression to diabetes specifically not described). ARR for progression to diabetes ranged from 1.6% to 7.1%. RR reduction for progression to diabetes in intervention, cf. control scenario=31–55%. Observed NNT for 1 year to avoid one case of diabetes=14.2 to 62.5. In meta-analysis, RR of progression to diabetes in intervention, cf. control arms=0.54 (95% CI 0.42 to 0.70).*
Selph, 2015	6 studies	Intervention associated with lower risk of progression to diabetes: meta-analysis RR=0.55 (95% CI 0.43 to 0.70). Similar results when Da Qing study (23-year follow-up) excluded: RR=0.53 (0.44 to 0.63).
Shellenberg, 2013	7 studies	Intervention associated with lower risk of progression to diabetes at 1 year (meta-analysis RR=0.35, 95% CI 0.14 to 0.85; four studies), 4 years (RR=0.56, 0.48 to 0.64; 2 studies), 6 years (RR=0.47, 0.34 to 0.65; 3 studies), and 10 years (RR=0.80, 0.74 to 0.88; one study). Da Qing study not included in meta-analysis, but noted that intervention associated with lower rates of progression to diabetes at 6 and 20 years, in this study.
Stevens, 2015	16 studies	In network meta-analysis (incorporating 16 lifestyle vs placebo/standard care studies) lifestyle intervention associated with lower risk of progression to diabetes: HR=0.65 (95% CI 0.56 to 0.74).
*Yoon, 2013*	*7 studies*	*T2DM incidence ranged from 3% to 46% in the intervention groups, cf. 9.3% to 67.7% in the control groups. Significantly lower T2DM incidence associated with intervention observed in 5/7 studies (RR reduction 28.5% to 64.7%). In the sixth study, lower diabetes incidence observed in the intervention vs control scenario at 1-year follow-up, but not at 3-years or 5-years. In the seventh study, intervention effect was observed only in the per protocol analysis (ie, no effect observed in intention to treat analysis).*
*Glycaemic control*		
Ashra, 2015	16 studies	No significant impact of intervention, cf. control condition observed at 12–18 months: net FPG difference=−0.06 mmol/L (95% CI −0.11 to 0.00; 16 trials). Significant impact observed at >18 months: net FPG difference=−0.07 mmol/L (−0.13 to −0.02).
	10 studies	No significant impact of intervention, cf. control condition observed at 12–18 months (net 2h-OGT difference=−0.28 mmol/L, 95% CI −0.57 to 0.00; 10 trials), or >18 months (difference=−0.52 mmol/L, −1.05 to 0.01; 7 studies).
Balk, 2015	6 studies	Intervention associated with reversion to normoglycaemia: meta-analysis summary RR=1.53 (95% CI 1.26 to 1.71).
	18 studies	At follow-up closest to 1 year, summary net change in FPG associated with intervention vs control condition=−0.12 mmol/L (−0.20 to −0.05; 17 studies). Net change in 2h-OGT=−0.48 mmol/L (−0.86 to −0.17; 11 studies) and net change in HbA_1c_=−0.08% (−0.12 to −0.04; 8 studies).
Cardonna-Morrell, 2010	9 studies	Authors concluded that the 1-year FPG outcomes across nine translational studies were in many cases similar to the DPP outcomes, but that effect size was too small to be clinically relevant.
	4 RCTs	No observed significant net impact of intervention vs control condition on 12-month FPG (difference=−0.19 mmol/L; 95% CI −0.44 to 0.06; 3 trials) or 2h-OGT (0.04 mmol/L, −0.49 to 0.42; 2 RCTs).
Gillett, 2012	5 systematic reviews; 9 RCTs	Authors conclude that most studies suggest intervention associated with reversion to normal glucose tolerance
Glechner, 2015	3 studies	At 1-year follow-up, intervention associated with significantly lower FPG (meta-analysis=−0.28 mmol/L; 95% CI −0.47 to −0.008), and 2h-OGT (−0.63 mmol/L, −1.08 to −0.18). Similar outcomes observed at 3-year follow-up (for FPG: −0.31 mmol/L; −0.48 to −0.15; for OGT: −0.68 mmol/L; 95% CI −1.03 to −0.34).
*Gong, 2015*	*7 studies*	*Mean net 2h-OGT difference associated with lifestyle vs control condition observed in meta-analysis=−0.65 mmol/L; 95% CI −1.35 to 0.05*
Norris, 2005	6 studies	The six studies that reported on HbA_1c_ were considered not to be representative of all nine studies identified for review. Results therefore not pooled, but effect of intervention ranged from 0.0% to −0.3%.
Orozco, 2008	7 studies	Meta-analysis of 6/7* studies demonstrated significant impact of intervention on FPG: net difference, cf. control condition=−0.19 mmol/L (95% CI −0.32 to −0.05).
	4 studies	Meta-analysis of 3/4* studies found no impact of intervention on 2h-OGT: net difference, cf. control condition=−0.23 mmol/L (−1.08 to 0.61).
*Santaguida, 2005*	*5 studies*	*Lifestyle intervention associated with significantly lower risk of progression to diabetes, or higher rate of reversion to normal glucose tolerance, in 4/5 trials reviewed (NB. number of studies considering glucose tolerance specifically not described.).*
Shellenberg, 2013	7 studies	Intervention associated with significantly lower FPG at 0.5–4 years follow-up: summary mean difference=−0.28 mmol/L, 95% CI −0.33 to −0.23. Authors concluded that data post-4 years follow-up insufficient to draw conclusions.
	5 studies	Intervention associated with significantly lower 2h-OGT at 1–4 years follow-up: summary mean difference=−0.54, −1.06 to −0.02. Again, data at post 4 years follow-up considered insufficient to draw conclusions.
	3 studies	No significant difference in HbA1c observed between intervention and control groups at 1–3 years follow-up: summary mean difference=−0.10, −0.22 to −0.01.
Zheng, 2015	12 studies	Intervention associated with significantly lower FPG, cf. control condition: mean difference=−0.22 mmol/L (95% CI −0.25 to −0.18; 9 studies). Also noted that the intervention effect increased with intervention duration, with the effect among the subgroup receiving the longest interventions (≥2 years duration) demonstrating the highest subtotal effect: mean difference for this subgroup=−0.24 mmol/L (−0.43 to −0.05; 12 studies).

Synthesis outcomes related to glycaemia are listed for each review, as relevant, alongside the number of primary studies drawn on in the associated syntheses. Italicised entries are those assigned AMSTAR scores <8, excluded from sensitivity analyses.

*Da Qing study not included in either meta-analysis due to cluster randomisation.

ARR, absolute risk reduction; DPP, Diabetes Prevention Programme; DPS, Diabetes Prevention Study; FPG, fasting plasma glucose; HbA1c, glycated haemoglobin; IRR, incidence rate ratio; NNT, number needed to treat; RCT, randomised controlled trial; RR, relative risk; T2DM, type 2 diabetes mellitus; 2h-OGT, 2-hours oral glucose tolerance.

#### Glycaemic control

Additional measures of glycaemia considered included measures of fasting glucose (seven reviews: seven meta-analyses), 2-hour oral glucose tolerance (2h-OGT; seven reviews: seven meta-analyses), HbA1c (three reviews; two meta-analyses, one narrative synthesis) and reversion to normoglycaemia (three reviews; one meta-analysis, two narrative syntheses) (see table 3). For fasting glucose measures, all but one meta-analysis suggested that interventions were associated with net reductions in glycaemia at follow-up. The lack of effect observed in one review may have been due to the study inclusion/exclusion criteria, which required that the primary studies were translations of the major diabetes prevention RCTs (ie, the DPP, Da Qing study, DPS or IDPP) into routine practice (and thus excluded the major larger-scale, longer term trials that tend to dominate the analyses in most reviews).^16^ This review similarly found no impact of the intervention on 2h-OGT,^16^ again out of keeping with the findings of other reviews. Only one other review observed no effect on 2h-OGT.^25^ This analysis was recently published and included a larger number of trials than the other reviews.

The clinical relevance of the effect sizes was queried in one review.^16^ Effect sizes were typically in the range 0.1–0.3 mmol/L for fasting glucose and 0.2–0.7 mmol/L for 2h-OGT (see table 3). A narrative synthesis of net intervention effect on HbA1c suggested this ranged from 0.0 to 0.3 percentage points,^35^ in keeping with corresponding meta-analysis outcomes that indicated intervention effects of −0.08% (95% CI −0.12 to −0.04) and −0.10% (−0.22 to −0.01).^27^ ^38^ Again, exclusion of studies with AMSTAR scores ≤7 (n=2) had little impact on general outcomes, which were largely consistent across reviews.

### Intervention effects—secondary outcomes

#### Behaviour change

Three reviews reported on the effects of interventions on dietary and/or physical activity behaviours (see table 4). Results were reportedly generally variable, with significant impact on behaviour noted in some primary studies only.^16^ ^28^ ^38^ One review noted that intervention benefit appeared to be greater where behavioural change was more pronounced and suggested that a relatively long intervention duration may be requisite for sustained behaviour change and sustained intervention impact on clinical parameters.^28^

**Table 4:** Secondary outcomes

Author, publication date	Number of studies included in synthesis	Outcomes
*Dietary and physical activity behaviours*		
Cardona-Morrell, 2010	3 studies	3/12 studies reviewed reported on changes in fat and fibre intake. Substantial improvements demonstrated in one trial only, which reported half the participants meeting fibre and total fat intake goals, and a third achieving saturated fat goal.
Gillett, 2012	8 studies	Authors concluded that adherence to lifestyle measures could be problematic and that compliance was variable. Noted that benefits of intervention greatest among those with the highest compliance and highest lifestyle target achievement. In one study (the DPS),^1^ strong inverse correlation between progression to diabetes and achievement of lifestyle targets noted. Suggested that relatively long duration of intervention (eg, 4 years of DPS) potentially necessary for lasting intervention impact.
Schellenberg, 2013	4 studies	Authors concluded that most studies reported positive effects on physical activity and dietary intake. However, results not always statistically or clinically significant or sustained after end of active intervention.
*Measures of adiposity*		
Ashra, 2015	20 studies	Pooled mean weight difference observed in intervention, cf. control arms of 20 RCTs at 12–18 months=−1.57 kg (95% CI −2.28 to −0.86). Weight change difference at >18 months (n=11 RCTs)=-1.26 kg (−2.35 to −0.18).
*Baker, 2011*	*6 studies*	*In the 6/7 studies for which impact on BMI could be calculated, endline BMI differences between intervention and control conditions consistently favoured the intervention, but effect sizes were small: range −0.05 kg/m^2^ (observed in IDPP) to −0.43 kg/m^2^ (observed in VIP). No tests for differences between conditions reported.*
Balk, 2015	24 studies	All studies observed net weight loss associated with intervention, of between 0.2% and 10.5% of initial body weight (summary net change=−2.2%, 95% CI −2.9 to −1.4)
Cardonna-Morrell, 2010	4 studies 2 studies	Meta-analysis outcomes suggest significant mean weight loss at 12 months associated with intervention: summary difference=−1.82 kg (95% CI −2.7 to −0.99) Pooled mean waist circumference measurement reduction significantly greater in treated vs control groups: mean difference=−4.6 cm (−5.8 to −3.4)
Gillett, 2012	5 systematic reviews, 9 RCTs	No summary weight change outcomes reported, but authors note that there was a tendency for weight to be regained soon after end of intervention. This did not occur in one study (DPS), and it was hypothesised that the duration of intervention (DPS=4 years) may be relevant to persistence of weight change.
Glechner, 2015	3 studies	Meta-analysis results suggest net mean weight difference associated with intervention, cf. control condition at 1 year=−2.44 kg (95% CI −3.45 to −1.43). Results consistent at 3 years: net weight difference=−2.45 kg (−3.56 to −1.33).
Norris, 2005	6 studies	At 1-year follow-up, the pooled estimate from four studies suggested additional weight loss of 2.8 kg (95% CI 4.7 to 1.0) in intervention, cf. control scenario, and net difference in BMI (three studies)=−1.3 kg/m^2^ (−1.9 to −0.8). At two-year follow-up, the net weight difference associated with intervention, cf. control scenario=−2.6 kg (−3.3 to −1.9; 3 studies).
Orozco, 2008	7 studies	Meta-analysis results suggested net BMI reduction associated with intervention=−1.1 kg/m^2^ (95% CI −2.0 to −0.2; 6 studies). Results for weight also indicated additional weight loss in the intervention group: summary net change=−2.7 kg (−4.7 to −0.7; 7 studies). No significant between-group difference observed for waist–hip ratio: summary difference=−0.01 (−0.02 to 0.01; 4 studies).
Schellenberg, 2013	8 studies	Authors state that most studies reported positive effects on body composition. However, results not always significant or sustained after end of active intervention.
*Yoon, 2013*	*5 studies*	*Two studies showed significant reduction in BMI associated with intervention, cf. control scenario (SLIM study: −0.36±1.47 kg/m^2^ in the intervention group, 0.08±1.80 in the control group, p=0.014; DPS study: −1.3±1.9 kg/m^2^ in the intervention group, −0.3±2.0 in the control group, p=<0.0001). In these and two further studies, significant weight loss observed in intervention groups in preintervention and postintervention comparisons. Significant weight increase observed in intervention and control groups of IDPP, but further details not reported.*
*Microvascular disease*		
Balk, 2015	1 study	The Da Qing study reported a reduction in severe retinopathy at 20-year follow-up associated with intervention, cf. control condition (HR=0.53, 95% CI 0.29 to 0.99). Limited evidence suggested no significant effects on nephropathy or neuropathy.
Schellenberg, 2013	1 study	The Da Qing study reported no effect on nephropathy or neuropathy at 20-year follow-up. However, incidence of severe retinopathy was 47% lower in intervention, cf. control participants. Authors commented that loss to follow-up was high and that many participants did not have formal retinal examinations. Hence, they considered the strength of evidence insufficient to draw conclusions.
*Macrovascular disease*		
Balk, 2015	2 studies	Authors commented that there was no consistent pattern in cardiovascular mortality outcomes. The Da Qing study observed no difference at 20-year follow-up (HR 0.83; 95% CI 0.48 to 1.40). In the DPP, no significant effect on cardiovascular mortality was observed at 3-year follow-up (RR 0.50; 0.09 to 2.73).
Gillett, 2012	4 studies	Authors concluded that studies with long durations of follow-up demonstrated disappointing CVD outcomes.
Hopper, 2011	2 studies	Non-significant trend towards reduction in cardiovascular mortality in meta-analysis of Da Qing study (20-year follow-up) and DPP (2.8-year follow-up) studies (RR 0.70, 95% CI 0.46 to 1.07).
Schellenberg, 2013	2 studies	No differences in CVD event rates between intervention and control groups noted at 10-year follow-up of DPS (RR=1.02, 95% CI 0.73 to 1.42), or the 6-year or 20-year follow-ups of the Da Qing study (at 6-year follow-up, HR=0.96; 0.76 to 1.44; at 20 years, HR=0.98, 0.71 to 1.37). Authors conclude that strength of evidence is insufficient to determine whether lifestyle interventions impact on CVD event rates.
*Yoon, 2013*	*2 studies*	*No differences in CVD event or mortality rates between intervention and control groups noted at 20-year follow-up of Da Qing study (for event rates, HR=0.98; 95% CI 0.71 to 1.37; for mortality rates, HR=0.83, 0.48 to 1.40). Only small number of CVD events observed at the three-year follow-up of the IDPP (n=4 in the intervention group, 2 in the control group).*
*Quality-adjusted life years*		
*Yoon, 2013*	*0 studies*	*Investigated and noted that no primary study reported on quality-adjusted life years.*
*All-cause mortality*		
Balk, 2015	3 studies	The 23-year follow-up data from the Da Qing study were indicative of lower risk of mortality in the intervention vs control arms (HR=0.71; 95% CI 0.51 to 0.99). This effect was restricted to women and not significant at earlier time points. No similar effect was observed at the 20-year follow-up or for men. No impact on all-cause mortality was observed at the 3-year follow-up of the DPP or 10-year follow-up of the DPS.
Hopper, 2011	4 studies	No impact of lifestyle intervention on all-cause mortality observed in meta-analysis (RR 0.81, 95% CI 0.61 to 1.09). (Studies considered=DPS 10-year follow-up, Da Qing study 20-year follow-up, DPP 2.8-year follow-up, IDPP 2.5-year follow-up).
Orozco, 2008	4 studies	Authors commented that all-cause mortality rates were comparable between the intervention and control groups. (Studies considered=Da Qing study 6-year follow-up, DPP 2.8-year follow-up, IDPP 2.5-year follow-up and the 2-year follow-up of a regional UK-based study).
*Yoon, 2013*	*1 study*	*Of studies reviewed, only the Da Qing study reported on mortality rate. No significant difference in overall mortality rate between the intervention and control group observed at 20-year follow-up (HR 0.96, 95% CI 0.65 to 1.41).*

Results relating to secondary outcomes are listed for each review, as relevant, alongside the number of primary studies drawn on in the associated syntheses. Italicised entries are those assigned AMSTAR scores <8, excluded from sensitivity analyses.

BMI, body mass index; CVD, cardiovascular disease; DPP, Diabetes Prevention Programme; DPS, Diabetes Prevention Study; DPS, Diabetes Prevention Study; IDPP, Indian Diabetes Prevention Programme; RCT, randomised controlled trial; RR, relative risk; VIP, Vasterbotten Intervention Programme.

#### Adiposity

Ten reviews considered measures of adiposity, which included weight change (seven reviews: six meta-analyses, one narrative synthesis), BMI (three reviews: two meta-analyses, one narrative synthesis), waist circumference and waist–hip ratio (one meta-analysis related to each) and body composition generally (two narrative syntheses) (see table 4). The six meta-analyses considering net weight difference associated with intervention at 1–2 years follow-up all suggested significant intervention effect, of ∼1–3 kg where reported in absolute terms (five studies). The associated narrative synthesis indicated that weight tended to be regained postend of intervention and that in the one case this did not occur (the DPS); this may have been attributable to relatively long intervention duration (4 years).^28^

BMI analysis outcomes were in keeping with the weight change results, with meta-analyses reporting significant net loss of ∼1.1–1.3 kg/m^2^ associated with intervention, and narrative synthesis also favouring intervention and noting a small effect size. Significant intervention effect was observed for waist circumference (−4.6 cm; 95% CI −5.8 to −3.4), whereas none was observed for waist–hip ratio (−0.01, −0.02 to 0.01), but these analyses drew on only two and four primary studies, respectively.^16^ ^36^ Two narrative syntheses were excluded from sensitivity analysis based on AMSTAR scores, and this had little impact on the generally consistent results.

#### Microvascular disease

Little evidence related to microvascular disease was identified (see table 4). Two reviews (both with AMSTAR scores >7) noted that only the Da Qing study had considered this and had reported associations between the intervention and protection against retinopathy (but not nephropathy or neuropathy), at 20-year follow-up.^27^ ^38^ However, these findings were reportedly limited by loss-to-follow-up and limited use of formal retinal examinations.^38^

#### Macrovascular disease

Five reviews provided narrative reports of intervention effect on cardiovascular disease including cardiovascular mortality (see table 4). Four reviews considered two primary studies each (the Da Qing study and DPP, DPS or IDPP).^27^ ^31^ ^38^ ^40^ No intervention effect on events and/or mortality was observed at the 6-year or 20-year follow-up of the Da Qing study, 3-year follow-up of the DPS or IDPP or 2.8-year DPP follow-up. An impact of the Da Qing study on cardiovascular mortality was observed for women only at 23-years follow-up. The fifth review concluded generally that cardiovascular disease outcomes at long durations of follow-up were disappointing.^28^ Only one review was excluded in sensitivity analysis.^40^

#### Quality-adjusted life years

Quality-adjusted life years were considered in one review only, and no relevant primary studies were identified (see table 4).^40^

#### All-cause mortality

Four reviews considered all-cause mortality, drawing on data from the Da Qing study, DPP, DPS, IDPP and a regional UK-based study (Oldroyd *et al*, 2006) (see table 4).^27^ ^31^ ^36^ ^40^ ^42^ A meta-analysis of the 10-year follow-up DPS data, 20-year follow-up Da Qing study data, 2.8-year follow-up DPP data and 2.8-year follow-up IDPP data indicated no intervention effect on mortality.^31^ Again intervention effect was only observed for women at the 23-year follow-up of the Da Qing study (HR 0.71, 95% CI 0.51 to 0.99).^27^

### Subgroup outcomes

#### Age

Six reviews considered the impact of age on intervention effect on incident diabetes (see table 5). Meta-regression was undertaken in three reviews (using data from 4, 11 and 18 studies, respectively).^25^ ^32^ ^33^ None observed an age effect. One narrative report indicated that significant within-study age-effects were observed for the DPP and DPS (greater intervention effect among older age groups).^27^ This DPP effect was not reported in a fifth review that considered the DPP only, but this review was excluded from sensitivity analysis in view of its AMSTAR score of 4.^37^

**Table 5:** Subgroup outcomes

Author, publication date	Number of studies included in synthesis	Outcomes
*Age*		
Ashra, 2015	18 studies	Meta-regression using data from 18 RCTs suggested study-level mean age did not impact on T2DM incidence, weight, glycaemia FPG and OGT (number of studies relevant to each outcome unclear). Similarly, study age-based inclusion criteria were not found to be associated with outcomes.
Balk, 2015	2 studies	Age effect considered for incident diabetes only. Discussion based on reported within-study subgroup analyses. Noted that DPP and DPS reported intervention had significantly greater impact on diabetes incidence in older age groups.
Merlotti, Morabito and Pontiroli, 2014	11 studies	In meta-regression using data from lifestyle intervention studies, no significant impact of age on cumulative incidence of diabetes observed.
Merlotti, Morabito, Ceriani and Pontiroli, 2014	4 studies	In meta-regression using data from lifestyle intervention studies, no significant impact of age on cumulative incidence of diabetes observed.
*Santaguida, 2005*	*1 study*	*The DPP study found no effect of age on the efficacy of the intervention in reducing progression to diabetes.*
Zheng, 2015	12 studies	In a stratified analysis of groups 40–55 and ≥55 years, no significant net effect on FPG observed in younger group (mean difference=−0.27 mmol/L, 95% CI −0.60 to 0.05). Effect observed for ≥55 years group (mean difference=−0.19 mmol/L, −0.22 to −0.15, p<0.05).
*Gender*		
Ashra, 2015	19 studies	A 1 unit increase in study-level baseline percentage of males was associated with a 3% higher incidence of T2DM (p=0.022), and borderline significantly associated with 0.05 kg weight gain (p=0.054), in those receiving intervention, cf. usual care. No impact on glycaemia observed.
Balk, 2015	2 studies	Sex differences considered for incident diabetes only. Discussion based on reported within-study subgroup analyses. Noted that sex differences investigated within DPP and DPS, but no significant effect on diabetes incidence detected.
Glechner, 2015	4 studies	Meta-analysis results for diabetes incidence at 1 year: for men, RR=0.53 (95% CI 0.26 to 1.10); for women, RR=0.71 (0.31 to 1.64); no difference by gender (p=0.61). Similar results at 3 years: for men RR=0.70 (0.53 to 0.91); for women RR=0.51 (0.35 to 0.75); no difference by gender (p=0.20). Da Qing study had the longest follow-up (6 years) and detected no significant difference in impact of intervention, between men and women.
	3 studies	In meta-analysis of body weight outcomes: similar additional mean weight reductions associated with intervention observed for males and females at 1 year (−2.29 kg (−5.22 to −0.76) and −2.65 kg (−4.23 to −1.07), respectively; p=0.74). At 3 years, additional mean weight reduction associated with intervention was −2.78 kg (−4.00 to −1.57) for males, and −0.6 kg (−3.43 to 2.24) for females; p=0.16.
	3 studies	In meta-analysis of glycaemia outcomes: at 1 year, males and females had similar mean reductions in FPG and 2h-OGT associated with intervention (for FPG, mean difference=−0.45 mmol/L (−1.10 to 0.19) and −0.26 mmol/L (−0.46 to −0.06), respectively; p=0.57; for 2h-OGT, mean difference=−0.77 mmol/L (−1.55 to 0.01) and −0.56 mmol/L (−1.12 to 0.00), respectively; p=0.67). Three-year follow-up outcomes were similar: for FPG, mean difference=−0.40 mmol/L (−0.58 to −0.21) and −0.08 mmol/L (−0.39 to 0.24), for males and females, respectively, p=0.09; for 2h-OGT, mean difference=−0.78 mmol/L (−1.33 to 0.24) and −0.62 mmol/L (−1.07 to −0.17), respectively, p=0.65.
*Santiguida, 2005*	*1 study*	*The DPP study found no effect of sex on the efficacy of intervention in reducing progression to diabetes.*
Selph, 2015	1 study	Noted that the Da Qing study detected significantly lower risk of all-cause mortality (HR 0.71; 95% CI 0.51 to 0.99) and CVD mortality (HR 0.59; 0.36 to 0.96) among intervention vs control participants, for females only, at 23-year follow-up. No significant effect of intervention observed among males. No clear explanation for disparity, but hypothesised potentially due to relatively poor compliance among males.
*Ethnicity*		
Ashra, 2015	13 studies	Study-level percentage of non-white participants not significantly associated with incidence of T2DM, weight change or glycaemia.
Balk, 2015	1 study	Discussion based on reported within-study subgroup analyses. Noted that differences by ethnicity considered in DPP, and no significant difference in effect of intervention detected.
*Modesti , 2016*	*8 studies*	*Meta-analysis demonstrated lower* *rates of incident diabetes among Asian participants assigned to intervention, cf. control condition: OR=0.55; 95% CI 0.44 to 0.70. No participants of other ethnic backgrounds reviewed.*
*Santaguida, 2005*	*1 study*	*Noted that the DPP study found no effect of ethnicity on the efficacy of intervention in reducing the progression to diabetes.*

Synthesis outcomes related to subgroups of interest are listed for each review, as relevant, alongside the number of primary studies drawn on in the associated syntheses. Italicised entries are those from reviews assigned AMSTAR scores <8, excluded from sensitivity analyses.

CVD, cardiovascular disease; DPP, Diabetes Prevention Programme; DPS, Diabetes Prevention Study; FPG, fasting plasma glucose; OGT, oral glucose tolerance; RCT, randomised controlled trial; RR, relative risk; T2DM, type 2 diabetes mellitus.

Two reviews considered the impact of age on continuous measures of glycaemia. No effect was observed in a meta-regression considering impact on fasting glucose (involving data from 14 primary studies) or 2h-OGT (10 studies).^25^ However, stratified analysis of pooled data from 12 studies suggested no effect of intervention in those aged 40–55 years, but a significant effect among those ≥55 years, with effect size=−0.19 mmol/L (95% CI −0.2 to −0.15).^41^

#### Gender

Four reviews investigated the impact of gender on incident diabetes (see table 5).^25^ ^27^ ^29^ ^37^ One considered four primary studies and noted no gender differences in either 1-year or 2-year follow-up meta-analysis results.^29^ In contrast, analysis of data from 19 studies in a second review suggested that each 1-unit increase in study-level baseline percentage of men was associated with a 3% higher diabetes incidence.^25^ Two narrative reviews (one excluded from sensitivity analysis) reported on within-study gender differences only, and no differences were observed in the two primary studies discussed.^27^ ^37^

No gender impact was observed when continuous measures of glycaemia were considered (two reviews).^25^ ^29^ Review of pooled individual-level data from three primary studies suggested no impact of gender on weight outcomes,^29^ but again a study-level review suggested that each percentage increase in the proportion of male participants was borderline significantly associated with 0.05 kg net weight gain.^25^

As above, one study noted the differential morbidity outcomes among men and women at the 23-year follow-up of the Da Qing study.^39^ The authors commented that lower intervention compliance among men may be relevant.

#### Ethnicity

Few data relating to effect of ethnicity were identified (see table 5). Two studies (one with AMSTAR score <8) noted only that the DPP identified no differences in incident diabetes by ethnicity.^27^ ^37^ One review of 13 primary studies noted that the percentage of non-white participants was not associated with diabetes incidence, weight change or glycaemia, at study level.^25^ A fourth review (with AMSTAR score <8) considered Asian participants only and noted that lifestyle interventions were associated with lower progression to diabetes in this population.^34^

#### Nature of trial

As mentioned above, most (13/19) reviews considered RCTs only, and in the remaining reviews, RCTs contributed most of the data considered. Consideration of efficacy versus effectiveness was therefore not possible, but one review included ‘translational’ studies only (whether RCT or not), and this was the only review to find no impact of lifestyle intervention on glycaemia.^16^ It was required that the interventions considered in the primary studies of this review were all delivered in routine clinical practice settings. They were of relatively short duration (1–48 months, median 32 weeks), and the studies also had relatively short-term follow-up (4–60 months, median 12 months). The authors of this review noted that intervention duration could be a mediator of intervention effect, and another review similarly suggested that intervention duration could be relevant to durability of effect.

## Discussion

### Principal findings

We aimed to provide an overview of systematic reviews of the clinical effectiveness of lifestyle interventions for those at high risk of diabetes and identified 19 reviews that met our inclusion criteria. The data considered within these reviews were largely from explanatory trials, and it was clearly demonstrated that lifestyle interventions can positively impact on diabetes incidence, although the size of effect on continuous measures of glycaemia was modest. Consistent impact on weight outcomes was also apparent, but one narrative synthesis noted that weight tended to start to be regained after the end of the intervention. There was also a suggestion that this weight regain could be avoided if the intervention was sufficiently lengthy (4 years in the example given).^28^ Relatively few behavioural outcome data were available, but these suggested that intervention effect on dietary and physical activity behaviours was more variable.^16^ ^28^ ^38^ There were also indications that behaviour change was associated with intervention effect on clinical parameters, and again it was suggested that the duration of intervention may be important for sustained impact on behaviour and therefore clinical outcomes.^28^ Very few data were available for the additional outcomes considered: microvascular and macrovascular disease, quality-adjusted life years and cardiovascular and all-cause mortality. There was some evidence that the unusually long duration (6-year) intervention assessed in the Da Qing Study impacted on the cardiovascular and all-cause morality of women, at 23-year (but not 20-year) follow-up. This intervention also appeared to impact on retinopathy at 20 years. However, there are some concerns about these retinopathy data,^38^ and the reviews considered suggested no impact on cardiovascular disease or mortality has been observed in any other trial to-date. Only a minority of reviews considered subgroups. Most synthesis outcomes suggested no impact of age on diabetes incidence or other measures of glycaemia, but there were reports of within-study effects of age within the DPP and DPS^27^ and an age effect on continuous measures of glycaemia in a stratified analysis.^41^ In all cases greater intervention impact was reported for higher age groups. In analyses of individual-level data, no effect of gender was observed for diabetes incidence, other measures of glycaemia or weight. There were no obvious indications of an effect of ethnicity, but this had been very little considered.

### Strengths and weaknesses of study

We carried out a wide, thorough and systematic search for relevant information and identified a large number of reviews for consideration, most of which were recent. However, there are several limitations of overviews that are relevant here, including the representation of primary studies in more than one review, and the reliance on individual review methodologies, with associated inability to consider the quality of the primary studies in detail, or study outcomes not investigated in the reviews themselves. In particular, there were few data available regarding subgroups. There was also limited availability of vascular end point data, linked to the short-term follow-up in most primary studies. We have not systematically searched for more recent primary studies not included in the reviews considered here. However, the latest update from the DPP is in keeping with the results reviewed,^43^ as are the results of additional recent trials,^44^ ^45^ including trials based in routine practice, where clinical benefits were found to be modest.^46^ ^47^ We excluded non-English language publications that we were unable to access (see figure 1). Whether these studies would otherwise have met our inclusion criteria is unclear.

### Findings in relation to previous work

Concern about the potentially limited impact of lifestyle interventions for the population with intermediate hyperglycaemia, on vascular disease and mortality end points, has been raised previously,^48–51^ particularly as these outcomes are likely to be those that most trouble patients and account for most of the direct health costs associated with diabetes treatment.^52^ Although data related to these outcomes are limited, they are derived from trials with the longest and most intensive interventions and relatively long durations of follow-up. It is therefore unlikely that impact on these outcomes would be observed following shorter interventions delivered in more routine care environments (ie, interventions more likely to be applied in practice), given that the relative impact of such interventions on intermediate outcomes is low and perhaps more transient.^16^ ^28^ Similarly transient effect of behaviour change interventions has been observed in the context of overweight/obesity management per se, even at reasonably short-term follow-up.^53^ ^54^ Additional reasons to be cautious generalising outcomes from explanatory behaviour change trials to those achievable in routine practice include the likely relatively high motivation of trial participants and the typical intervention resourcing and fidelity achieved in trials versus routine practice settings.^55^ Population-level impact would also require that sufficient numbers of individuals eligible to access the intervention are identified, offered the intervention, and choose to participate. In many settings, new resources would be required to identify relevant patients for a lifestyle intervention programme, and few data regarding intervention uptake are available to-date. There has similarly been little investigation into potentially negative aspects of such interventions. For example, there is the potential for participant disengagement—both with the specific intervention and lifestyle change more generally—if anticipated programme outcomes are difficult to achieve and/or maintain.^56^ The possibility of staff disengagement has been raised previously.^57^

### Implications for future research, policy and practice

Despite the lack of demonstrated effectiveness of lifestyle interventions for diabetes prevention—and apparent modest impact on outcomes of interest even under trial conditions—the option of population-wide intervention roll-out is currently being discussed in several countries. NHS England has recently started to implement a programme across England, but so far as we are aware, no other country has adopted a national programme as yet, perhaps due to the outstanding clinical and cost-effectiveness concerns. Cost-effectiveness remains a concern as to-date cost-effectiveness studies have been reliant on data from the studies reviewed above and thus subject to the attendant limitations. Cost-effectiveness studies have also typically not factored-in costs for associated requisite activities such as diabetes risk-assessment services, which the WHO has warned could overwhelm primary care.^4^ Rigorous evaluation of any programme that is implemented could help address the evidence gap relating to effectiveness of DPPs.

In view of the anticipated limited impact of lifestyle interventions for diabetes prevention on diabetes and cardiovascular disease risk burdens, many have recently commented that a broader approach to these issues is overdue.^50^ ^51^ ^58^ National and international guidance and policy documents have for many years advocated for relevant environmental change through widespread actions across many policy domains and associated legislative and fiscal commitment.^4^ ^8^ ^59^ ^60^ For example, action on information, marketing and pricing for tobacco, food and alcohol has been recommended, as well as sensitivity to the health impacts of agriculture, transport, education and urban planning policy. There is good evidence to support some of these wider policy options that would reduce the accessibility of products and lifestyles associated with overweight/obesity, diabetes and cardiovascular risk and promote access to the opposite.^61–63^ Renewed focus on such options may be useful.

## Conclusions

There have been many recent systematic reviews of DPPs, consistently demonstrative that lifestyle interventions for populations at high diabetes risk can reduce or delay risk of progression to diabetes. However, the reviewed data are overwhelmingly from RCTs. Where intervention duration and setting have been considered, outcomes from shorter interventions in routine practice settings appear more variable. In keeping with this, recent in-practice trials have achieved modest results. Some countries are considering national roll-out of lifestyle interventions for diabetes prevention. Thorough and early evaluation of any such programme would be useful. Additional approaches will be required if we are to impact on the global diabetes burden.
