# Supplementary material for: Clinical impact of lifestyle interventions for the prevention of diabetes: an overview of systematic reviews
Source: BMJ Open. 2016 Dec 21;6(12):e013806. doi: 10.1136/bmjopen-2016-013806 (PMC5223710; doi:10.1136/bmjopen-2016-013806)
Supplement: supplementary appendices [file bmjopen-2016-013806supp_appendices.pdf]

Appendix 1: Example database search strategy

Strategy used to search Embase database via Ovid

1. *impaired glucose tolerance/
2. *non insulin dependent diabetes mellitus/pc [Prevention]
3. *diabetes mellitus/pc [Prevention]
4. *glucose intolerance/
5. intermediate hyperglyc?emia.tw.
6. subdiabetic hyperglyc?emia.tw.
7. sub-diabetic hyperglyc?emia.tw.
8. nondiabetic hyperglyc?emia.tw.
9. non-diabetic hyperglyc?emia.tw.
10. borderline diabetes.tw.
11. borderline diabetic.tw.
12. borderline HbA1c.tw.
13. borderline hyperglyc?emia.tw.
14. (borderline h?emoglobin adj A1c).tw.
15. borderline A1c.tw.
16. prediabetes.tw.
17. prediabetic.tw.
18. pre-diabetes.tw.
19. pre-diabetic.tw.
20. impaired glucose tolerance.tw.
21. impaired fasting glucose.tw.
22. impaired fasting glyc?emia.tw.
23. impaired glucose regulation.tw.
24. impaired glyc?emic function.tw.
25. prediabetes state.tw.
26. prediabetes states.tw.
27. prediabetic state.tw.
28. prediabetic states.tw.
29. pre-diabetes state.tw.
30. pre-diabetes states.tw.
31. pre-diabetic state.tw.
32. pre-diabetic states.tw.
33. glucose tolerance impairment.tw.
34. latent diabetes.tw.
35. latent diabetic.tw.
36. prediabetes stage.tw.
37. prediabetic stage.tw.
38. pre-diabetes stage.tw.
39. pre-diabetic stage.tw.
40. intermediate glycemic control.tw.
41. impaired glucose sensitivity.tw.
42. glucose dysregulation.tw.
43. 1 or 3 or 4 or 5 or 6 or 7 or 8 or 9 or 10 or 11 or 12 or 13 or 14 or 15 or 16 or 17 or 18 or 19 or 20 or 21 or 22 or 23 or 24 or 25 or 26 or 27 or 28 or 29 or 30 or 31 or 32 or 33 or 34 or 35 or 36 or 37 or 38 or 39 or 40 or 41 or 42
44. diet/
45. exercise/
46. diet therapy/
47. physical activity/
48. preventive health service/
49. behavior modification/
50. nutrition/
51. diabetes education/

52. behaviour change/
53. *prevention/
54. lifestyle modification/
55. sport/
56. *lifestyle/
57. harm reduction/
58. *risk reduction/
59. *health education/
60. nutritional counseling/
61. behavior therapy/
62. *health promotion/
63. dietetics.tw.
64. dietician.tw.
65. nutritionist.tw.
66. lifestyle intervention.tw.
67. life-style intervention.tw.
68. non-pharmacological intervention.tw.
69. Prevention intervention.tw.
70. Preventative intervention.tw.
71. Preventive intervention.tw.
72. resistance training.tw.
73. aerobics.tw.
74. workout.tw.
75. strength training.tw.
76. weight training.tw.
77. physical conditioning.tw.
78. lifestyle intervention.tw.
79. work-out.tw.
80. life-style intervention.tw.
81. risk reduction.tw.
82. community based intervention.tw.
83. community based programme.tw.
84. community based programme.tw.
85. prevention programme.tw.
86. prevention programme.tw.
87. 44 or 45 or 46 or 47 or 48 or 49 or 50 or 51 or 52 or 53 or 54 or 55 or 56 or 57 or 58 or 59 or 60 or 61 or 62 or 63 or 64 or 65 or 66 or 67 or 68 or 69 or 70 or 71 or 72 or 73 or 74 or 75 or 76 or 77 or 78 or 79 or 80 or 81 or 82 or 83 or 84 or 85 or 86
88. meta-analys:.mp.
89. search:.tw.
90. review.pt.
91. 88 or 89 or 90
92. 43 and 87 and 91

Appendix 2: Excluded studies

List of studies excluded at stage of full text review, categorised by reasons for exclusion

<u>**POPULATION**</u>

Low primary study n-numbers

Aguiar EJ, Morgan PJ, Collins CE, Plotnikoff RC, Callister R. Efficacy of interventions that include diet, aerobic and resistance training components for type 2 diabetes prevention: A systematic review with meta-analysis. Int J Behav Nutr Phys Act 2014;11(1):2.

Galani C, Schneider H. Prevention and treatment of obesity with lifestyle interventions: review and meta-analysis. Int J Public Health 2007;52(6):348-59.

Johnson M, Jones R, Freeman C, et al. Can diabetes prevention programmes be translated effectively into real-world settings and still deliver improved outcomes? A synthesis of evidence. Diabet Med 2013;30(1):3-15.

Rawal L, Tapp R, Williams E, et al. Prevention of Type 2 Diabetes and Its Complications in Developing Countries: A Review. Int J Behav Med 2012;19(2):121-33.

Population characteristics do not meet inclusion/exclusion criteria

Angermayr L, Melchart D, Linde K. Multifactorial lifestyle interventions in the primary and secondary prevention of cardiovascular disease and type 2 diabetes mellitus--a systematic review of randomized controlled trials. *Ann Behav Med 2010*;40:49–64.

Bouaziz W, Schmitt E, Kaltenbach G, Geny B, Vogel T. Health benefits of endurance training alone or combined with diet for obese patients over 60: a review. Int J Clin Pract 2015:69(10);1032-1049.

Boyers D, Avenell A, Stewart F, et al. A systematic review of the cost-effectiveness of non-surgical obesity interventions in men. Obes Res Clin Pract 2015;9(4):310-327.

Chapman J, Qureshi N, Kai, J. Effectiveness of physical activity and dietary interventions in South Asian populations: a systematic review. *Br J Gen Pract* 2013;63:E104–E114.

Lirussi, F. The global challenge of type 2 diabetes and the strategies for response in ethnic minority groups. *Diabetes Metab Res Rev* 2010;26:421–432.

Robertson C, Archibald D, Avenell A, et al. Systematic reviews of and integrated report on the quantitative, qualitative and economic evidence base for the management of obesity in men. *Health Technol Assess* 2014;18:1–424.

Udell JA, Cavender MA. Bhatt DL, et al. Glucose-lowering drugs or strategies and cardiovascular outcomes in patients with or at risk for type 2 diabetes: a meta-analysis of randomised controlled trials. Lancet Diabetes Endocrinol 2015;3(5):356-366.

Uthman OA, Hartley L, Rees K, et al. Multiple risk factor interventions for primary prevention of cardiovascular disease in low- and middle-income countries. *Cochrane Database Syst Rev* 2015;8:CD011163.

Wingo BC, Carson TL, Ard J. Differences in weight loss and health outcomes among African Americans and whites in multicentre trials. *Obes Rev* 2014;15:46–61.

INTERVENTION

Physical activity only

Aune D, Norat T, Leitzmann M, et al. Physical activity and the risk of type 2 diabetes: A systematic review and dose-response meta-analysis. Eur J Epidemiol 2015;30(7):529-542.

Sasser H. Take Two Sneakers and Call Me in the Morning: Drugs, Exercise, and Mortality. Integrative Medicine Alert 2014;17(5):54-56.

COMPARATOR

Comparator = medication use

Yuen A, Sugeng Y, Weiland TJ, Jelinek GA. Lifestyle and medication interventions for the prevention or delay of type 2 diabetes mellitus in prediabetes: a systematic review of randomised controlled trials. Aust N Z J Public Health 2010;34(2):172-78.

OUTCOMES

Insufficient follow-up duration

Ali MK, Echouffo-Tcheugui J, Williamson DF. How effective were lifestyle interventions in real-world settings that were modeled on the Diabetes Prevention Program? *Health Aff (Millwood)*, 2012;31(1):67–75.

Samuel-Hodge CD, Johnson CM, Braxton DF, Lackey M. Effectiveness of Diabetes Prevention Program translations among African Americans. *Obes Rev* 2014;15(S4):107–124.

Outcomes of interest not considered

Lin JS, O'Connor E, Evans CV, Senger CA, Rowland MG, Groom HC. Behavioral Counseling to Promote a Healthy Lifestyle in Persons With Cardiovascular Risk Factors: A Systematic Review for the US Preventive Services Task Force. *Ann Intern Med* 2014;161:568–578.

STUDY TYPE

Systematic review criteria not met

Abate N, Chandalia M. The impact of ethnicity on type 2 diabetes. J Diabetes Complications 2003;17(1):39-58.

Allende-Vigo MZ. Diabetes mellitus prevention. Am J Ther 2015;22(1):68-72.

Angelo JB, Huang J, Carden D. Diabetes prevention: A review of current literature. *Advanced Studies in Medicine* 2005;5:250–259.

Ardisson Korat AV, Willett WC, Hu FB. Diet, Lifestyle, and Genetic Risk Factors for Type 2 Diabetes: A Review from the Nurses' Health Study, Nurses' Health Study 2, and Health Professionals' Follow-Up Study. *Curr Nutr Rep* 2014;3:345–354.

Burnet DL, Elliott LD, Quinn MT, Plaut AJ, Schwartz MA, Chin MH. Preventing diabetes in the clinical setting. *J Gen Intern Med* 2006;21:84–93.

Chiasson JL. Prevention of Type 2 diabetes: fact or fiction? *Expert Opin Pharmacother* 2007:8(18):3147–3158.

Deen D. Metabolic syndrome: Time for action. Am Fam Physician 2004;69:2875–2888.

Dyson PA. Addressing non-communicable disease at the population level: A focus on diabetes. *Diabetes Manag* 2014;4:153–163.

Fujii RK, Junqueira M, Restrepo M, Turatti LA. Metformin and intensive lifestyle intervention for pre-diabetes – systematic review of efficacy. Value Health 2015;18(3):A55-A56.

Haw JS, Tantry S, Vellanki P, Pasquwl FJ. National Strategies to Decrease the Burden of Diabetes and Its Complications. Curr Diab Rep 2015;15(9):65.

Hussain A, Claussen B, Ramachandran A, Williams R. Prevention of type 2 diabetes: A review. *Diabetes Res Clin Pract* 2007;76:317–326

Jackson L. Translating the diabetes prevention program into practice: A review of community interventions. *Diabetes Educ* 2009;35:309–320.

Kanat M, DeFronzo RA, Abdul-Ghani MA. Treatment of prediabetes. World J Diabetes 2015;6(12):1207-1222.

Paulweber B, Valensi P, Lindstrom J, et al. A European evidence-based guideline for the prevention of type 2 diabetes. *Horm Metab Res* 2010;42:S3–S36.

Lauritzen T, Borch-Johnsen K, Sandbaek A. Is prevention of Type-2 diabetes feasible and efficient in primary care? A systematic PubMed review. *Prim Care Diabetes* 2007;1:5–11.

Lukacova-Zib I, Gopalakrishnan G. Therapeutic options for the prevention of type 2 diabetes mellitus in the metabolic syndrome. *Mt Sinai J Med* 2010;77:524–532.

Madden SG, Loeb SJ, Smith CA. An integrative literature review of lifestyle interventions for the prevention of type II diabetes mellitus. *J Clin Nurs* 2008;17:2243–2256.

Maez L, Erickson L, Naumuk L. Diabetic education in rural areas. Rural Remote Health 2014;14(2):1-7.

Melander A. Review of previous impaired glucose tolerance intervention studies. *Diabet Med* 1996;13:S20–2.

Mohan V, Seedat YK, Pradeepa R. The rising burden of diabetes and hypertension in Southeast Asian and African regions: Need for effective strategies for prevention and control in primary health care settings. *Int J Hypertens* 2013;409083.

Mudaliar S. Choice of early treatment regimen and impact on beta-cell preservation in type 2 diabetes. *Int J Clin Pract* 2013;67:876–887.

Palermo A, Maggi D, Maurizi AR, Pozzilli P, Buzetti R. Prevention of type 2 diabetes mellitus: Is it feasible? *Diabetes Metab Res Rev* 2014;30:4–12.

Pronk NP, Remington PL. Combined Diet and Physical Activity Promotion Programs for Prevention of Diabetes: Community Preventive Services Task Force Recommendation Statement. *Ann Intern Med* 2015;163(6):465–468.

Qvigstad E. Prevention of type 2 diabetes: An overview. *Tidsskrift for den Norske Laegeforening* 2004;124(23):3047–3050.

Satterfield DW, Volansky M, Caspersen CJ, et al. Community-Based Lifestyle Interventions to Prevent Type 2 Diabetes. *Diabetes Care* 2003;26(9):2643–2652.

Senechal M, Slaght J, Bharti N, Bouchard DR. Independent and combined effect of diet and exercise in adults with prediabetes. *Diabetes Metab Syndr Obes* 2014;7:521–529.

Shrestha P, Ghimire L. A review about the effect of life style modification on diabetes and quality of life. *Glob J Health Sci* 2012;4:185–190.

Tuduri XM. Can type 2 diabetes mellitus be prevented by means of lifestyle changes or pharmacological measures in patients with glucose intolerance? *FMC Formacion Medica Continuada en Atencion Primaria* 2007;14:442.

Walicka M, Chomiuk T, Filipiak KJ, et al. Type 2 diabetes prevention. Experts' Group position paper endorsed by the Polish Cardiac Society Working Group on Cardiovascular Pharmacotherapy. Kardiologia Polska 2015;73(10):949-957.

Walker KZ, O'Dea K, Gomez M, Girgis S, Colagiuri R. Diet and exercise in the prevention of diabetes. *J Hum Nutr Diet* 2010;23:344–352.

Wilding JPH. The importance of weight management in type 2 diabetes mellitus. *Int J Clin Pract* 2014;68:682–691.

Yamaoka K, Tango T. Efficacy of Lifestyle Education to Prevent Type 2 Diabetes A meta-analysis of randomized controlled trials. *Diabetes Care* 2005;28(11):2780–2786.

Thomas GN, Jiang CQ, Taheri S, et al. A systematic review of lifestyle modification and glucose intolerance in the prevention of type 2 diabetes. *Curr Diabetes Rev* 2010;6(6):378–387.

Yamaoka K, Tango T. Efficacy of lifestyle education in preventing type 2 diabetes: An updated version. *Salud i Ciencia* 2009;17:29–33.

Protocol for incomplete review

Lagisetty P, Priyadarshini S, Landgraf J. Evaluating the impact culturally tailored interventions for diabetes prevention. PROSPERO 2015:CRD42015016914 Available from http://www.crd.york.ac.uk/PROSPERO/display_record.asp?ID=CRD42015016914

Qin Q, Almeida F, Estabrooks P, Pardo K, Karlsson C. How effective are diabetes prevention lifestyle interventions in reducing glycaemia levels? A systematic review. PROSPERO 2014:CRD42014010429 Available from http://www.crd.york.ac.uk/PROSPERO/display_record.asp?ID=CRD42014010429

OTHER

Abstract with insufficient information to determine whether inclusions/exclusion criteria met

Bradley E, Bautista L, McBride P. Absolute glucose changes with lifestyle modification in impaired glucose tolerance. Journal of the American College of Cardiology 2010;55:A58.

Fujii RK, Junqueira M, Restrepo M, Turatti LA. Metformin and intensive lifestyle intervention for pre-diabetes-systematic review of efficacy. Value Health 2015;18(3):A55-A56.

Abstract superseded by full paper

Zheng L, Wang G, Li J, et al. Efficacy of exercise-only versus exercise-diet in the prevention of type 2 diabetes among pre-diabetic population: A meta-analysis. J Am Coll Cardiol 2015;66(16):C128.

Harreiter J, Glechner A, Gartlehner G, et al. Sex-specific differences in prevention of type 2 diabetes mellitus: A systematic review and meta-analysis. *Diabetologia* 2014;57:S120.

Zhang X, Imperatore G, Thomas W, et al. Effect of lifestyle interventions on glucose regulation among adults without impaired glucose tolerance or diabetes: A systematic review. Diabetes 2015;64:A425-A426.

Yoon U, Kwok LL, Magkidis A. Efficacy of lifestyle interventions in reducing diabetes incidence in patients with impaired glucose tolerance: A systematic review of randomized controlled trials. *BMC Proceedings*, 2012;6:P28.

Review updated/superseded by another included review

Dunkley AJ, Bodicoat DH, Greaves CJ, et al. Diabetes prevention in the real world: Effectiveness of pragmatic lifestyle interventions for the prevention of type 2 diabetes and of the impact of adherence to guideline recommendations - A systematic review and meta-analysis. Diabetes Care 2014;37(4):922-33

National Institute for Health and Care Excellence. Preventing type 2 diabetes: risk identification and interventions for individuals at high risk. NICE, 2012. Available at: https://www.nice.org.uk/guidance/ph38. Accessed: June 2016.

Gillies CL, Abrams KR, Lambert PC, et al. Pharmacological and lifestyle interventions to prevent or delay type 2 diabetes in people with impaired glucose tolerance: Systematic review and meta-analysis. Br Med J 2007;334(7588):299-302.

Norris SL, Kansagara D, Bougatsos C, Fu R, US Preventive Services Task Force. Screening adults for type 2 diabetes: A review of the evidence for the U.S. preventive services task force. Ann Intern Med 2008;148(11):855-68.

Foreign language paper – unable to access sufficient content to assess versus inclusion/exclusion criteria

Chiasson JL. Is prevention of diabetes possible? *Journees annuelles de diabetologie de l'Hotel-Dieu* 2003:91–97.

Chiasson JL. Screening and prevention of diabetes. *Perspective infirmiere: revue officielle de l'Ordre des infirmieres et infirmiers du Quebec* 2006;4:18–21.

Kasuga M. [Prediction and prevention of NIDDM] *Nihon Rinsho* 1997;55:S345–348.

Kosaka K. Primary prevention of type 2 diabetes mellitus according to lifestyle intervention- the process and for the future. *Nippon Rinsho* 2002;60(S9):52–99.

Kumagai S, Sasaki H. Evaluation of health promotion program for patients with glucose intolerance. *Nippon Rinsho* 2005;63(S2):649–653.

Kuzuya H, Tsujii S. Lifestyle interventions for preventing non insulin-dependent diabetes mellitus. *Nippon Rinsho* 1997;55:349–353.

Nunoi K, Fukui T, Sato Y, et al. Management of IGT education program at a hospital and its primary prevention effect on diabetes mellitus. *Nippon Rinsho* 2005;63(S2):582–587.

Okayama A, Nohara M. Diabetes prevention program of healthy Japan 21. *Nippon Rinsho* 2002;60(S1):613–619.

Sakane N. Japan Diabetes Prevention Program. *Nippon Rinsho* 2005;63(S2): 488–492.

Sauvanet JP. Altering lifestyle and treating obesity, preventing type 2 diabetes? *Soins; la revue de reference infirmiere*, 2004;684:19–24.

Sumida Y. [An approach to the prevention of type 2 diabetes in industrial healthcare]. *Nihon Rinsho* 2005;63(S2):588–596.

Tawaramoto K, Kaku K. Treatment for patients with impaired glucose tolerance. *Nippon Rinsho* 2005;63(S2):423–427.

Thivolet C. Pre-diabetes type 1. Evaluation of current clinical trials. *Journees annuelles de diabetologie de l'Hotel-Dieu*, 1997:201–205.

Tuomilehto J. Modeling of primary prevention of the development of type 2 diabetes. *Przeglad lekarski* 2006;63(S4):3–6.

Tuomilehto J, Lindstrom J. The principal trials for prevention of type 2 diabetes have now confirmed that type 2 diabetes can be prevented by life style changes. Prevention of type 2 diabetes by life style. *Journees annuelles de diabetologie de l'Hotel-Dieu*, 2003:77–89.

Uusitupa M. Exercise and diet as targeted treatments of metabolic syndrome. *Duodecim; laaketieteellinen aikakauskirja*, 2001;117:621–630.

Wepner U. [Prevention by life style intervention and medication. To run away from type 2 diabetes]. *MMW Fortschr Med* 2004;146:16.

Yoshida T, Umekawa T. [Treatment of obese patients with IGT]. *Nihon Rinsho* 2005;63(S2):552–556.

Appendix 3: Characteristics of included reviews

The inclusion/exclusion criteria of each included review, outcomes of interest (as per review questions) assessed, and quality assessment (AMSTAR score) are summarised, alongside the review publication dates, the publication dates of the reviewed primary studies, and the number of reviewed studies, publications and participants pertaining to investigation of lifestyle interventions. Italicised entries are those assigned AMSTAR scores <8, excluded from sensitivity analyses.

T2DM: type 2 diabetes mellitus; FPG: fasting plasma glucose; 2h-OGT: 2-hour oral glucose tolerance; HbA1c: glycated haemoglobin; NR: not reported; WHO: World Health Organization; RCT: randomised controlled trial; IGT: impaired glucose tolerance; IFG: impaired fasting glucose; ADA: American Diabetes Association; BMI: body mass index; CVD: cardiovascular disease; QALY: quality adjusted life year

**Author, publication date**	**Publication dates of reviewed primary studies**	**Number of studies/ number of publications reviewed**<br>**Participants (n)**	**Inclusion criteria**<br>**Exclusion criteria**	**Outcomes assessed**	**AMSTAR score**
Ashra, 2015	2007-2015	36/49<br>n=12,407	*Inclusion*: Adults (>18 years) at high risk of T2DM (= obese, sedentary lifestyle, family history of T2DM, older age, metabolic syndrome, impaired glucose regulation, pre-diabetes, elevated diabetes risk score); follow-up ≥ one year; published in English<br>*Excluded*: Studies where >10% of the population had established T2DM	T2DM incidence; glycaemia (FPG, 2h-OGT, HbA1c); body weight	9
Baker, 2011	*Not referenced*	*7/95*<br>*n=5,825*	*Inclusion: Adults (definition NR) diagnosed as dysmetabolic or with IGT, as per WHO criteria; any intervention involving exercise training, physical activity and/or nutritional advice; RCTs; published in English*<br>*Exclusion: Pre-existing T2DM*	*T2DM incidence*	*6*
Balk, 2015	1991-2015	53/104<br>n=23,497	*Inclusion*: Participants of any age with pre-diabetes (as per measures of glycaemia or diabetes risk assessment, not defined further) or metabolic syndrome; combined dietary and physical activity intervention with at least two contact sessions (in-person or virtual) over ≥3 months conducted in any outpatient setting; RCTs and prospective non-randomised comparative studies with ≥30 participants/group, and single-group intervention studies with ≥100 participants; follow-up ≥6 months; published in any	T2DM incidence; glycaemia (FPG, 2h-OGT); reversion to normoglycaemia; body weight; diabetes complications; all-cause	9

			language<br>*Exclusion*: Single-food or other supplement dietary intervention; intervention with pharmacological component	mortality	
Cardonna-Morrell, 2010	1990-2009	12/13<br>n=5,728	*Inclusion*: Adults (definition NR) with IGT/IFG (definition NR) and/or T2DM risk factors such as obesity or family history; dietary ± physical activity intervention of ≥3 months duration delivered through routine healthcare services; translational research studies of RCT, before/after, cohort, or interrupted time series design; published in English language; studies including participants with diabetes were included so long as outcomes for those without pre-existing diabetes were reported separately<br>*Exclusion*: Interventions limited to education materials or pharmacological agents	T2DM incidence; glycaemia (FPG, 2h-OGT, HbA1c); physical activity; fibre and fat consumption	10
Gillett, 2012	1999-2006	14/9<br>n=5,875	*Inclusion*: Participants (demographics NR) with IGT/IFG (definition NR); weight loss interventions incorporating exercise and/or dietary modification components; comparator group of standard or non-intensive lifestyle treatment; RCTs of at least two years duration and systematic reviews of RCTs<br>*Exclusion*: Pre-existing T2DM	T2DM incidence; glycaemia (FPG, 2h-OGT, IGT, HbA_{1C}); body weight; diet and physical activity behaviours	8
Glechner, 2015	1993-2011	7/21<br>n=2,170	*Inclusion*: Participants (demographics NR) with IFG/IGT as per any current/ previous ADA or WHO definition; comparator group exposed to usual treatment or glucose lowering agent [NB. studies with usual treatment comparator group analysed separately]; RCTs; published in English or German<br>*Exclusion*: Studies of women with prior gestational diabetes only	T2DM incidence; glycaemia (FPG, 2h-OGT); body weight; diabetes complications; all-cause mortality	9
Gong, 2015	*1997-2011*	*9/9*<br>*n=2,968*	*Inclusion: Adults (definition NR) with IGT (definition NR); intervention to delay or prevent T2DM; RCTs; follow-up at least one year; published in any language*<br>*Exclusion: Pre-existing T2DM; no glycaemia outcome reported*	*Glycaemia (FPG, 2h-OGT)*	*7*
Hopper, 2011	1974-2010	4/7<br>n= 3,495	*Inclusion*: Adults (definition NR) with IGT (= 2h-OGT 7.8-11.0 mmol/L) or IFG (= FPG 6.1-6.9 mmol/L); diet and/or physical activity or pharmacological intervention; RCTs; follow-up at least one year; published in English<br>*Exclusion*: n <100	T2DM incidence; all-cause and cardiovascular mortality	8

Merlotti, 2014a	1979-2012	11/12<br>n= 4,581	*Inclusion*: Participant demographics NR; diagnosis of IGT, IFG, or obesity, or history of gestational diabetes; any intervention with the aim of T2DM prevention; experimental and observational studies; published in any language<br>*Exclusion*: None reported	T2DM incidence	8
Merlotti, 2014b	2006-2013	4/4<br>n= 2,993	*Inclusion*: Participants of any age; diagnosis of IGT, IFG, or obesity, or history of gestational diabetes; diabetes incidence reported as outcome; intervention and follow-up of any duration; randomised or non-randomised controlled studies; published in any language<br>*Exclusion*: None reported	T2DM incidence	10
Modesti, 2016	*1997-2014*	*8/8*<br>*n=2,721*	*Inclusion: Asian individuals (definition NR) with IGT or IFG diagnosis; studies reporting the effect of lifestyle interventions on incidence of T2DM; RCTs; intervention and follow-up of any duration; publication language requirements NR*<br>*Exclusion: Pre-existing T2DM; duplicate publications*	*T2DM incidence*	*6*
Norris, 2005	1979-2003	9/25<br>n=5,168	*Inclusion*: Adults (>18 years) with IGT or IFG (as defined by criteria at time of primary study); interventions with weight loss/control as a primary stated goal; published or unpublished RCTs; follow-up at least one year; published in any language<br>*Exclusion*: Intervention = advice to increase exercise level without further physical activity component; pharmacological interventions	Glycaemia; body weight; BMI; cardiovascular events; cardiovascular- and all-cause- mortality	11
Orozco, 2008	1997-2007	8/25<br>n=5,956	*Inclusion*: Participants of any age with at least one risk factor for T2DM (= IGT, IFG, gestational diabetes history, hypertension, family history of T2DM, obesity, dyslipidaemia, high risk ethnic group); physical activity ± diet intervention of $\geq$6 months duration; RCTs; published in any language<br>*Exclusion*: Pharmacological therapy as intervention or control	T2DM incidence; glycaemia (IGT, IFG); body weight; BMI; waist circumference; waist-to-hip ratio	11
Santaguida, 2005	*1997-2003*	*5/10*<br>*n= NR*	*Inclusion: Participants (demographics NR) with IGT or IFG (definition NR); interventions with lifestyle, behavioural or pharmaceutical component; RCTs; minimum 6 month follow-up; publication post-1978; published in English language*<br>*Exclusion: systematic reviews, other reviews, editorials, letters, unpublished material,*	*Glycaemia; diabetes complications; CVD incidence; all-cause mortality*	*4*

			practice guidelines		
Schellenberg, 2013	1992-2011	9/9<br>n= NR	*Inclusion*: Adults (>18 years) at high risk of T2DM (defined as metabolic syndrome, pre-diabetes, insulin resistance, IFG, IGT, syndrome X, dysmetabolic syndrome X, or Reaven syndrome); lifestyle interventions with physical activity and dietary components, plus any additional component (e.g. counselling, smoking cessation, behaviour modification); intervention ≥3 months duration; follow-up ≥6 months duration; RCTs; published in English language<br>*Exclusion*: None reported	T2DM incidence; glycaemia (FPG, HbA1c); body composition; physical activity and dietary behaviours; diabetes complications	10
Selph, 2015	2006-2013	6/10<br>n=NR	*Inclusion*: Adults (demographics NR) with IGT or IFG (definition NR); lifestyle intervention with the aim of improving glycaemic control; RCTs, controlled observational studies or systematic reviews; publication language requirements NR<br>*Exclusion*: Pre-existing T2DM in participants; children, adolescents and pregnant women	T2DM incidence	10
Stevens, 2015	1979-2013	14/18<br>n=NR	Inclusion: Adults (>18 years) with IFG (FPG 6.1-6.9 mmol/L and 2h-OGT <7.8 mmol/L) or IGT (FPG <7.0 mmol/L and 2h-OGT 7.8-11.1 mmol/L); lifestyle, pharmacological or surgical weight loss intervention; RCTs; publication language requirements NR<br>*Exclusion*: None reported	Time to T2DM diagnosis	8
Yoon, 2013	*1992-2009*	*7/25*<br>*n=5,663*	*Inclusion: Participants (demographics NR) with IGT (definition NR); lifestyle intervention tested versus 'control group' (not defined); interventions ≥6 months duration; RCTs; publication language requirements NR*<br>*Exclusion: study design unclear, observational studies; studies that included patients with IFG or established diabetes; pharmacological intervention*	*T2DM incidence; body weight; BMI; all-cause mortality; QALYs*	*7*
Zheng, 2015	2001-2013	12/12<br>n = 4,021	*Inclusion:* Adults > 40 years with pre-diabetes as per WHO (1998) or ADA (2003) criteria; interventions including exercise or exercise plus diet components; any study design; publication language requirements NR<br>*Exclusion:* History of T2DM; pharmacological intervention	Glycaemia (FPG)	9

Appendix 4: Characteristics of the populations and lifestyle interventions studied in each included review

Details of the participants and interventions considered in each review are summarised. The participant characteristics include those of participants in study arms additional to lifestyle and control arms, where relevant. Italicised entries are those assigned AMSTAR scores <8, excluded from sensitivity analyses.

F/U: follow-up; NR: not reported; BMI: body mass index; SD: standard deviation; IQR: interquartile range; RCT: randomised controlled trial

Author, publication date	Intervention Details	Intervention duration<br>Follow-up duration (F/U)	Participant baseline characteristics
**Ashra, 2015**	Interventions (n =36) included diet plus physical activity components (n =34), or physical activity only (n =2). In 12 cases, the physical activity intervention involved supervised sessions. Studies were generally conducted in either a primary care, outpatient or community setting. Two studies were additionally conducted in the work place and one study in a hospital setting.	Intervention duration NR<br>F/U: 12 weeks - 4 years	Mean age: 38-65 years; mean BMI: 25-37kg/m^2; % female: 34-100%; ethnicity data NR
Baker, 2011	*All studies (n = 7) involved combined diet and physical activity interventions. All of the physical activity components involved a supervised (n = 3) or unsupervised (n = 4) structured aerobic exercise programme. Three additionally incorporated supervised resistance training, and three incorporated occupational activity. Recommended activity levels ranged from 35 to 420 min/week. The dietary component involved individual dietary assessment and advice to decrease fat intake in all cases. Six studies prescribed portion control, five advised reductions in alcohol intake, four advised more fibre intake. All studies included initial face-to-face assessment, and group or individual counselling. All employed a team of specialist staff to deliver the interventions. The number of contact sessions in the first 12 months ranged from 6-32.*	*Intervention duration: 1-6 years*<br>*F/U NR*	*Mean age (± SD): 42±9 to 56±6 years; % male: 44%; three studies stated a minimum BMI between 24 and 27; authors suggested cohort was ethnically diverse*
**Balk, 2015**	All reviewed interventions (n = 66) had both diet and physical activity components. Twenty-seven were nominally based on the DPP or DPS, 42 involved weight loss goals, 16 involved individually-tailored diet plans, and 19 included dietary goals. Twenty-three involved individually tailored exercise plans, and 32 included exercise goals. Recommended physical activity levels ranged from ≥2 sessions of 20-30 min/week to 60	Intervention duration: 5-72 months<br>F/U NR (although inclusion criteria	Median age: 53.6 years; median % female: 65%; >75% of studies included overweight or obese participants; 74.5% of participants were of white

	min/day. Dietary advice included information on calorie, carbohydrate, fat and fibre goals. Sixty-one interventions lasted ≥6 months. Seven programmes were delivered entirely via internet, video, telephone or email. The remainder used face-to-face individual or group sessions, or both. Sessions were various combinations of trained diet and/or exercise counsellors, nurses, physicians, psychologists and trained laypersons. Sessions were delivered in healthcare and community settings. The median number of sessions was 15 (IQR 6.5-24.5). Median number of core sessions was 10 (6-16), and where applicable (n = 28) the median number of maintenance sessions was 6 (1.5-12).	required follow-up of ≥6 months)	ethnic background
**Cardonna-Morrell, 2010**	All studies (n=12) involved combined physical activity and dietary intervention. All dietary interventions were structured; half of the physical activity interventions were structured and half unstructured. The nature of the interventions was otherwise unspecified. All interventions were delivered in routine clinical settings including hospital outpatient departments, general practice and other community health services. Interventions were delivered by nurses or allied health professionals. Five to 54 sessions were delivered either in a group or individual setting.	Intervention duration: 1-48 months; median 8 months<br>F/U: 4-60 months; median 12 months	Age: 20-79 years; gender, BMI and ethnicity data NR
**Gillett, 2012**	Details of interventions assessed within five systematic reviews considered NR. All interventions (n=9) tested within the primary studies considered included both dietary and physical activity components. All included advice on dietary composition, several included specific calorie restriction advice and one recommended an isocaloric diet. The physical activity components included advice to increase overall activity levels (two studies), or advice to achieve physical activity goals (generally at least 150 min/week, but 20-30 min/week in one case, and 90 min/week in another). Several interventions utilised weight loss goals. Individual and/or group sessions were initially run at intervals from 3x/weekly to 3-4 monthly. Several interventions included lower intensity maintenance phases.	Intervention duration NR<br>F/U: 1.8-20 years	Age: 35-58.2 years; % female: 0-67.7%; mean BMI 24- 36 kg/m^2; ethnicity reported in some studies only: one study had 54.7% white participants, one involved only Japanese males, one only Japanese Americans
**Glechner, 2015**	Interventions (in n = 7 RCTs) included regular dietary advice and instructions for physical activity. Most were delivered on an individual basis with additional group sessions. One study involved the family of the participants, and two studies involved additional support from a dietician or medical staff. Three studies offered supervised physical activity	Intervention duration NR<br>F/U: 1-6 years	Mean age: 45-55 years; % male: 55%; mean BMI: 25.3-24.5 kg/m^2; authors report that the population was

	training programmes.		ethnically diverse
Gong, 2015	*Seven interventions included both dietary and physical activity components. Two were physical activity interventions only. Details of intervention content, delivery and intensity NR.*	*Intervention duration NR*<br>*F/U: 12-240 months; mean 58 months*	*Mean age: 54.3 years (range 45-65); mean BMI: 28.4kg/m^2 (range 24.6-31.2); gender and ethnicity data NR*
**Hopper, 2011**	All lifestyle interventions studied (n = 4) included diet and exercise components. Details on content, delivery and intensity NR.	Intervention duration: 2.5-6 years; mean 3.75 years<br>F/U: 2.8-6 years	Mean age: 52 years (range 45-64); % male: 47%; ethnicity and BMI data NR
**Merlotti, 2014a**	All eleven studies considered combined diet and physical activity interventions. Five studies reportedly involved physical activity sessions ranging from 20-30 min/week to 30-40 min/day. No further details of the additional physical activity components, dietary components, programme delivery or setting were reported.	Intervention duration NR<br>F/U: 1.5-13.4 years	Mean age: 33.4-58.4 years; mean BMI (±SD): 28.9±2.84 kg/m^2; gender data NR; authors reported that the majority of participants were Caucasian
**Merlotti, 2014b**	Three of the four studies reviewed considered combined diet and physical activity interventions. One study considered a single component physical activity intervention. Little information about the interventions, their delivery and setting were available.	Intervention duration NR<br>F/U: mean 3.8 ± 1.77 years	Mean age (±SD): 54.2±3.39 years; mean BMI: 33.0±1.61 kg/m^2; gender and ethnicity data NR
Modesti, 2016	*All interventions studied (n = 8) involved dietary and physical activity advice. Regarding physical activity, two studies encouraged self-monitoring, two involved use of pedometers, one involved a phone reminder service whereby participants were reminded to carry out 30 min of physical activity per day. Five studies generally encouraged regular physical activity and one recommended >30min daily brisk walking. Regarding diet, detailed specific advice regarding proportions of different nutrients was offered in five studies. One study provided resources on cooking, and a shopping tour, and three studies used a weight loss goal. Limited information about programme delivery was available, but some cases the programmes were delivered by various combinations of dieticians,*	*Intervention duration NR*<br>*F/U: 18-72 months*	*Age and gender data NR; in one study, minimum BMI threshold = 22; only Asian populations included*

	nurses, physical therapists and physicians.		
**Norris, 2005**	Eight of nine studies reviewed included combined diet and physical activity interventions; one did not include a physical activity component. Physical activity interventions varied from counselling and encouraging increased activity, to supervised sessions several times a week, and in one case a daily 2.5hr/day aerobic session as part of a residential programme. Regarding diet, seven studies advised caloric restriction, one advised an isocaloric diet with specific advice on fat and carbohydrate intake, and one involved carbohydrate restriction. The intensity and duration of session delivery varied from once session every three months for one year (i.e. four sessions in total), to 3x/week for six months (i.e. 78 total contacts).	Intervention duration: 4 weeks - 10 years<br>F/U: 1-10 years; mean 3.2 years	Mean age: = 51.2 years; mean BMI: 28.7 kg/m^2 (range 25.8-34.0); mean % female: 50%; authors reported variation in ethnicity across studies.
**Orozco, 2008**	All studies reviewed (n = 8) involved combined diet and physical activity interventions. Dietary advice included restricting fat and carbohydrate/calorie intake, increase fibre consumption, and portion control. In one study, advice was given to reduce meal sizes by 5-10%, depending on BMI. Physical activity interventions included supervised exercise programmes with an average of 150 min/week of brisk walking, cycling or jogging. Interventions were delivered in both group and individual format, by physiotherapists and dieticians. Total number of contacts ranged from 5 to 51.	Intervention duration: 1-6 years<br>F/U: 1-6 years; median 4 years	Mean age: 45-58 years; mean BMI: 25.8-35.9 kg/m^2; % female: 0-81%; reportedly mainly Asian and Caucasian populations
Santaguida, 2005	*All interventions (n = 5) included diet and physical activity components. Further information about intervention content, delivery and setting NR.*	*Intervention duration NR*<br>*F/U: 0.5-6 years*	*Age: 24-75 years; gender and BMI data NR; no summary ethnicity data, but reported that one study included only Japanese Americans and another only Europeans*
**Schellenberg, 2013**	All lifestyle interventions (n = 9) included diet and physical activity components. Six interventions specified weight loss goals. Dietary advice included calorie restriction (6 studies), general advice on (variably) fat, fibre, sugar, alcohol, carbohydrate, fruit and vegetable intake (7 studies), on established guidelines, and individualised dietary advice (4 studies). Most studies (n = 5) advised physical activity level of at least 150 min/week,	Intervention duration: 6 months – 6 years<br>F/U: 6 months - 20 years	Mean age: 44-85 years; % male: 0-69%; mean BMI (±SD): 26.2±3.9 to 38.3±5.9 kg/m^2; ethnicity reportedly varied across studies

	one advised any increase in activity levels and one advised achievement of 20-30 min 2-3x/week. Four interventions offered supervised activity sessions, three provided individual advice, and one provided incentivisation. Five involved both individual and group counselling; one only individual counselling, another only group counselling, and two regular telephone contact. Interventions were variably delivered by dieticians, exercise advisors, nurses, doctors and psychologists.		
**Selph, 2015**	All interventions (n = 6) included diet and physical activity components. Little information about the specifics of intervention content and delivery was provided, although frequency and duration of session delivery reportedly varied. One included a one-month stay at a 'wellness centre'.	Intervention duration = 5 years in one study; otherwise NR<br>F/U: 1-5 years	Mean age: 45-58.5 years; mean BMI: 24.2-32.6 kg/m^2; % female: 28-70%; no summary ethnicity data, but one study reported ethnicity = 73.5% white
**Stevens, 2015**	Fourteen interventions included diet and physical activity components. Further information about intervention content, delivery and setting NR.	Intervention duration NR<br>F/U: 1.5-9.4 years	Mean age: 38.7-63.8 years; mean BMI: 24.6-37.4 kg/m^2; gender data NR; authors reported population of heterogeneous ethnic background
Yoon, 2013	*All studies (n =7) involved combined diet and physical activity interventions. All dietary interventions included advice on dietary composition, and three included calorie restriction advice. Three studies advised at least 150/min physical activity per week, two advised any increase in physical activity, one provided general activity advice, and one provided a range of supervised physical activity sessions. Five studies included weight loss goals. Three provided individualised advice.*	*Intervention duration NR*<br>*F/U: 2.8-6 years*	*Mean age: 45-55; mean BMI: 24-35 kg/m^2; one study included only male participants, otherwise % female ranged from 21-68%; ethnicity data NR*
**Zheng, 2015**	Nine of the twelve interventions studied involved both exercise and physical activity components; three were physical activity-only interventions. Further information about intervention content, delivery and setting NR	Intervention duration NR<br>F/U: 0.5-4 years	Mean age: 53-63.6 years; gender, BMI and ethnicity data NR
